# 

**Published:** 1892-10

**Authors:** 


### HALL'S JOURNAL of HEALTH

ESTABLISHED 1854

CONTENTS

VOL. 39. No. 10.

| | PAGE. |
|---|---|
| The Cholera | 217 |
| Shocked into Silence | 219 |
| Nurses in the Sick Room | 220 |
| Summer Complaints | 223 |
| Healing in Precious Stones | 227 |
| Christopher Columbus | 230 |
| Asleep for Nine Years | 232 |
| A Simple Remedy for Sprains | 232 |
| Sulfonal in Insomnia | 233 |
| Ancient Hospitals | 234 |
| Objection to New Bread | 235 |
| Round Shoulders | 235 |
| Anti-Tobacco | 236 |
| Miscellaneous | 236 |
| Over and Over Again | 240 |

INDEX TO ADVERTISEMENTS OF HALF A PAGE AND OVER.

| | PAGE. |
|---|---|
| Dr. Jaegers | I |
| Christian Life | I |
| Magic Fuel Co. | II |
| The Rip Van Winkle Rocker | IV |
| Hall's Journal of Health | IV |
| Ayer's Sarsaparilla | V |
| Hall's Journal Premiums | VII |
| Washington Improvement Co. | VIII |
| Herba Vita | X |
| Royal Baking Powder | XI |
| Buffalo Lithia Water | XI |

